# Late-Onset Erotomanic Delusions, Cognitive Impairment, and Ventriculomegaly in an Older Adult Without Prior Psychiatric History: A Neuropsychiatric Case Report

**DOI:** 10.3390/brainsci16101046

**Published:** 2026-09-29

**Authors:** Edicer Ramírez-Rivera, Alexandra N. Burgos-Vélez

**Affiliations:** Department of Psychiatry, University of Puerto Rico, Medical Sciences Campus, P.O. Box 365067, San Juan, PR 00936-5067, USA; alexandra.burgos3@upr.edu

**Keywords:** late-onset psychosis, erotomania, de Clérambault syndrome, normal pressure hydrocephalus, ventriculomegaly, vascular cognitive impairment, neurocognitive disorder, decisional capacity, elder financial exploitation, geriatric psychiatry

## Abstract

**Highlights:**

**What are the main findings?**

- In a 75-year-old man with no psychiatric history, subacute erotomanic delusions occurred together with worsening cognitive screening performance (MoCA 23/30 to 17/30 over one month) and ventriculomegaly disproportionate to cortical atrophy. These findings co-occurred, and this single case does not establish how they are related.
- Acting on his delusional belief that he was in a relationship with a public figure, the patient sold his home and transferred approximately $200,000 to individuals impersonating the celebrity online, illustrating how an erotomanic syndrome and cognitive impairment together can produce catastrophic financial harm.

**What are the implications of the main findings?**

- Late-onset erotomanic delusions without prior psychiatric history should prompt structural neuroimaging and longitudinal cognitive follow-up and should not be assumed to represent an isolated primary psychiatric syndrome.
- Ventriculomegaly in this setting raises the question of a potentially reversible contributor, normal pressure hydrocephalus, which remained unconfirmed in this patient. Neuropsychiatric evaluation should be paired with formal decisional capacity assessment and protective legal measures considered where capacity assessment demonstrates significant impairment and less restrictive alternatives are insufficient.

**Abstract:**

Background: Late-onset psychosis (onset after age 60) in a patient with no prior psychiatric history is uncommon and frequently signals an underlying neurocognitive or structural brain disorder rather than a primary psychiatric illness. Erotomania, the delusional belief that an inaccessible or higher-status person is in love with the patient, has rarely been described together with cognitive impairment and ventriculomegaly. We describe a case in which these three features occurred together and set out the competing explanations for their co-occurrence. Methods: We report a single retrospective case, drawing on inpatient psychiatric and neurology consultation records, cognitive screening with the Montreal Cognitive Assessment (MoCA), head computed tomography, brain magnetic resonance imaging, and one-month outpatient follow-up. Written informed consent for publication was obtained from the patient during the hospitalization, and his capacity for that specific decision was assessed by the treating team. Results: A 75-year-old man with no psychiatric history developed subacute, fixed erotomanic delusions involving a public figure, with an illogical and circumstantial thought process, intermittent disorientation, and severely impaired judgment. Acting on these beliefs, he sold his home and transferred approximately $200,000 to individuals impersonating the celebrity online. Neuroimaging showed ventriculomegaly disproportionate to cortical sulcal prominence together with chronic small-vessel ischemic change. Neurology characterized the picture as borderline or early normal pressure hydrocephalus versus hydrocephalus ex vacuo with a superimposed neurocognitive disorder. The recommended diagnostic large-volume lumbar puncture was not performed, and no quantitative morphometric measurements were obtained, so normal pressure hydrocephalus remained unconfirmed. MoCA performance worsened from 23/30 at admission to 17/30 one month later, although both assessments were obtained during active psychosis and antipsychotic treatment. He was treated with low-dose antipsychotic medication and discharged with outpatient psychiatric and neurosurgical follow-up, and delusions and cognitive impairment persisted at short-term follow-up. Conclusions: New-onset erotomanic delusions in an older adult should prompt evaluation for an underlying neurocognitive or neurological disorder, including structural imaging for potentially reversible causes. This single case cannot establish the direction of the relationship among the erotomanic syndrome, the cognitive impairment, and the ventriculomegaly. Because impaired judgment in this setting can produce catastrophic financial harm, clinicians should pair neuropsychiatric evaluation with formal decisional capacity assessment.

## 1. Introduction

Late-onset psychosis, defined as psychotic symptoms emerging after age 60, is uncommon and is frequently secondary to an underlying medical or neurocognitive condition rather than a primary psychiatric disorder [1]. When psychosis presents de novo in an older adult without a prior psychiatric history, it warrants thorough medical and neurological evaluation before a primary psychotic disorder is diagnosed [2]. The International Psychogeriatric Association’s Psychosis in Neurodegenerative Disease working group has identified psychosis emerging late in life as a risk factor for later cognitive decline, which supports the view that late-onset psychotic symptoms may, in some patients, represent a prodromal phase of a neurocognitive disorder rather than a purely psychiatric event [3]. That association describes groups of patients, and it does not establish the sequence of events in any individual case.

Erotomania, also known as de Clérambault syndrome, is a delusional disorder in which the patient holds a fixed, false belief that another person, often of higher social status, is in love with them [4]. The forensic literature on erotomania has focused predominantly on the risk the deluded individual poses to the object of the delusion, through stalking, harassment, and occasionally violence [4,5]. Far less attention has been paid to the harm erotomania can cause the patient rather than others, and the intersection of erotomanic delusions with dementia has been described in only a handful of case reports [6].

Financial impairment is increasingly recognized as one of the earliest detectable signs of Alzheimer’s disease and related dementias, often preceding formal diagnosis by several years; a study using credit-bureau data from more than 80,000 Medicare beneficiaries demonstrated that adverse credit outcomes begin to increase as early as six years before a dementia diagnosis, with missed payments rising significantly in the years preceding clinical recognition [7]. The convergence of delusional thinking and impaired financial judgment therefore creates a high-risk scenario for catastrophic financial loss, particularly as cognitively vulnerable older adults become frequent targets of romance-scam and impersonation schemes conducted by telephone and social media.

Structural brain abnormalities, including ventriculomegaly and chronic small-vessel ischemic change, may accompany cognitive and behavioral change in older adults, but the relationship between these findings and new-onset psychiatric symptoms is often unclear. Ventriculomegaly may reflect normal pressure hydrocephalus (NPH), hydrocephalus ex vacuo from cortical volume loss, or an incidental finding of aging [8,9,10]. Distinguishing among these possibilities matters clinically because NPH is one of the few potentially reversible causes of cognitive decline [9]. Cerebral small-vessel disease, which commonly coexists with both, can independently produce executive dysfunction, behavioral change, and late-life psychotic symptoms, and its contribution is frequently difficult to separate from that of the other two.

We present the case of a 75-year-old man, a retired economist and financial consultant with no prior psychiatric history, who developed subacute erotomanic delusions and worsening cognitive performance in the context of ventriculomegaly, and who sold his home and transferred approximately $200,000 to individuals impersonating a celebrity he believed to be his romantic partner. Beyond the clinical description, we set out the competing explanations for the co-occurrence of these findings and their implications for financial decisional capacity and protective intervention. We do not claim to resolve which explanation is correct, and we explain why a single retrospective case cannot do so.

## 2. Case Presentation

### 2.1. Patient Information and History of Present Illness

The patient was a 75-year-old man, a retired economist and financial consultant with graduate-level education, who had been living independently and managing his own finances without prior psychiatric or substance use history. According to collateral history obtained from his daughter, he developed progressive cognitive and behavioral changes over approximately one month before hospitalization. He came to hold a fixed erotomanic delusion that he was in a romantic and marital relationship with a well-known American actress, reporting that he received messages from her through television broadcasts and social media and that intermediaries were facilitating their relationship.

Acting on this belief, he first used personal savings and then sold his home, transferring approximately $200,000 to individuals who contacted him by telephone and social media while impersonating the celebrity. Family members later reviewed his phone and confirmed messages and calls from a third party exploiting his mental state. He also solicited money from neighbors, stating that he needed to send it to his “partner.” After selling his home he moved into a rented apartment, where he exhibited disorganized behavior, spatial disorientation, agitation, and inappropriate requests for money from neighbors, and was subsequently evicted. In the days preceding presentation he was missing for approximately four days and was ultimately found at an airport, disheveled, combative, unbathed, and with unclear food intake.

He was brought involuntarily to the emergency department under the Puerto Rico Mental Health Code of 2000 (Act 408-2000) [11], the commonwealth’s involuntary psychiatric commitment statute, which permits hospitalization when mental illness renders a person a danger to themselves or others. In this case the risk was directed chiefly at himself, evidenced by severe financial self-endangerment, loss of housing, impaired self-care, and ongoing vulnerability to exploitation.

His past medical history was notable for hypertension, dyslipidemia, chronic small-vessel ischemic disease on prior imaging, and unilateral blindness of unclear etiology. He had no history of seizures, head trauma, or prior neurocognitive diagnosis. Home medications were losartan 100 mg daily, hydrochlorothiazide 25 mg daily, and simvastatin 10 mg nightly.

### 2.2. Emergency Department and Psychiatric Evaluation

On initial evaluation the patient denied any concern about his situation and did not believe that he required medical or psychiatric care, reflecting limited insight into the circumstances surrounding his presentation. Mental status examination revealed a thought process that was circumstantial and, at times, illogical and irrelevant. He endorsed a fixed delusional belief that he was in a romantic relationship with the actress and was sending her money. He was intermittently confused and disoriented to place and situation, with restricted, incongruent affect, poor concentration, and markedly impaired insight, judgment, and impulse control. He denied suicidal and homicidal ideation and auditory and visual hallucinations. He minimized or denied the events his daughter described, so his account of the preceding month was considered unreliable. He was medicated with intramuscular haloperidol 2.5 mg for agitation in the emergency department.

Head computed tomography obtained on admission showed diffuse prominence of the cortical sulci consistent with age-related involutional change, periventricular white-matter hypodensities consistent with chronic small-vessel ischemic disease, and, notably, ventriculomegaly disproportionate to the degree of cerebral volume loss, raising concern for hydrocephalus. He was admitted involuntarily to the inpatient psychiatric unit with a working diagnosis of unspecified psychosis, with major neurocognitive disorder and delirium secondary to a medical condition on the differential, given his psychosocial stressors (financial loss, poor social support) and the imaging findings.

### 2.3. Cognitive Screening and Neuroimaging

The Montreal Cognitive Assessment (MoCA) performed on hospital day 1 yielded a score of 23/30. The MoCA is a screening instrument and does not establish a diagnosis or a severity category, and the patient was in active psychosis at the time of testing. Neurology was consulted given the imaging findings and the concern for a structural process contributing to the neuropsychiatric presentation. Neurological examination revealed a patient who was awake and cooperative, oriented to person but inconsistently to place and time. Cranial nerves, motor bulk, tone, strength, and sensation were grossly intact, and reflexes were symmetric without pathologic signs. No tremor, rigidity, bradykinesia, or other extrapyramidal sign was documented at this examination. Gait, assessed in a subsequent addendum, was independent and largely preserved, with only mild difficulty in single-leg foot tapping and inability to reliably perform a tiptoe gait. There was no magnetic gait, no gait apraxia, and no urinary incontinence, so only the cognitive element of the Hakim and Adams triad was present.

Brain MRI without contrast, obtained the following day for further characterization, confirmed marked ventriculomegaly of the lateral ventricles disproportionate to cortical sulcal prominence, with periventricular and scattered deep white-matter T2/FLAIR hyperintensities (Figure 1). No restricted diffusion, midline shift, mass effect, or hemorrhage was identified. The radiological report described the ventricular system as prominent and out of proportion to the cortical sulci, gave a differential of central atrophy versus normal pressure hydrocephalus, and advised clinical correlation and further workup. It interpreted the white-matter changes as mild microangiopathic small-vessel disease. Periventricular signal change cannot be cleanly attributed on conventional sequences in a patient who has both ventricular enlargement and established small-vessel disease. The scattered distribution of the deep lesions supports a chronic ischemic contribution, but a transependymal component related to the ventriculomegaly cannot be excluded, and the appearance is probably mixed. This distinction was not formally adjudicated.

Neurology reviewed the CT and MRI before gait had been assessed, considered the appearance consistent with communicating hydrocephalus, and recorded an initial impression of most likely normal pressure hydrocephalus, an interpretation informed by current ACR appropriateness criteria for dementia imaging [8]. Gait was assessed later on the same evening, and the assessment was revised in an addendum. The patient ambulated independently with adequate gait, without magnetic gait or significant gait apraxia, and without urinary incontinence, so the Hakim and Adams triad was incomplete and cognitive and behavioral change predominated. Neurology therefore recharacterized the presentation as borderline or early normal pressure hydrocephalus versus hydrocephalus ex vacuo with a superimposed neurocognitive disorder [9,10]. Neurology reached this formulation after examining the patient, and it is the formulation used throughout this report.

No quantitative morphometric measurements were obtained. The Evans index, the callosal angle, and features of disproportionately enlarged subarachnoid-space hydrocephalus were not measured during the admission [12]. Neurology recommended formal review of the MRI with attention to the callosal angle and supportive morphologic findings, and that review was not completed before discharge. Definitive evaluation was also recommended via a diagnostic large-volume lumbar puncture, or tap test, with objective pre- and post-procedure assessment of gait and cognition [13]. Given the absence of inpatient neurosurgical services and ongoing psychiatric instability, outpatient neurosurgical evaluation was recommended instead.

Laboratory evaluation, including complete blood count, metabolic and hepatic panels, thyroid-stimulating hormone, vitamin B12, rapid plasma reagin, urine toxicology, and urinalysis, was unremarkable aside from a mild leukocytosis of 12.20 × 103/mcL with neutrophil predominance and nonspecific urinalysis abnormalities (Table 1). No infectious source was identified, and no antimicrobial treatment was given. These findings argue against a major metabolic or toxic contributor, although they do not exclude a superimposed delirium at the time of admission. Vital signs remained stable throughout admission.

### 2.4. Clinical Course and Treatment

Quetiapine, initiated at admission for psychosis, was discontinued on hospital day 2 because of concern that its sedating and hypotensive effects could increase fall risk in a patient with incompletely characterized hydrocephalus. Risperidone 0.5 mg daily was substituted as a lower-sedation alternative, and the dose was not changed during the admission.

At admission the patient was poorly cooperative, oriented to person but only partially to time, place, and situation, with an illogical and circumstantial thought process. His mental state on the unit improved over the following days. By hospital day 3 he was cooperative and maintained good eye contact, although he remained suspicious, and he was oriented to person and place and partially to situation, with a thought process that was coherent although at times illogical and irrelevant. The erotomanic delusion remained fixed, and he continued to describe paranoid ideation toward staff and family, with persistently poor insight, judgment, and impulse control. No tremor, rigidity, or bradykinesia was documented at any point during the admission. He consistently denied suicidal and homicidal ideation throughout the admission. He remained in the psychiatric unit until he no longer met the statutory criteria for continued involuntary hospitalization.

Written informed consent for publication was obtained from the patient during the hospitalization. It is important to consider that a patient’s capacity to provide consent is specific to the decision at hand and should not be inferred solely from their broader cognitive functioning. The treating psychiatric team therefore assessed his capacity for this particular decision at the time consent was obtained, using the standard framework of understanding, appreciation, reasoning, and expression of a choice [14]. He was able to understand what publication of a de-identified case report involves, to appreciate that it applied to him, to reason about the decision, and to express a consistent choice, and the team judged that he retained capacity for it. That judgment is compatible with the concern documented elsewhere in this report regarding his capacity to manage complex financial affairs, which is a different decision with a different threshold of complexity and consequence.

### 2.5. Outcome and Follow-Up

The patient was discharged on risperidone 0.5 mg orally at bedtime with outpatient psychiatric follow-up and a pending outpatient neurosurgical referral for consideration of diagnostic lumbar puncture. At psychiatric follow-up approximately one month after discharge, he remained on the same dose and reported ongoing use. Adherence was self-reported, and collateral was not available at that visit to corroborate it. There had been no meaningful change in psychotic symptom severity over the month. He continued to exhibit fixed erotomanic delusions with poor insight, minimized recent events, and perceived his family’s efforts to assist with financial management and government benefits as intrusive. Examination revealed intact orientation to person and place with disorientation to time and a persistently illogical, tangential thought process.

A bilateral resting tremor of the upper extremities was observed at this visit. No tremor had been documented on any examination before risperidone was started, including the neurology consultation. No examination was performed between discharge and this visit, so the onset of the tremor relative to antipsychotic exposure cannot be established. Formal extrapyramidal rating scales were not administered because there was no rigidity, no bradykinesia, and no other clinical sign of parkinsonism apart from the tremor.

Baseline collateral from his daughter had documented substantial deterioration in instrumental function before admission, including loss of independent financial management, sale of his home, eviction from subsequent housing, spatial disorientation, impaired self-care with poor hygiene and uncertain oral intake, and a four-day period during which he was missing. At follow-up collateral was unavailable, which limits formal comparison of basic and instrumental activities of daily living. The patient attended the appointment independently, arranged his own transportation, and had purchased food, indicating some preserved community functioning at one month. No standardized functional instrument was administered at either time point.

Repeat MoCA at this visit scored 17/30, six points lower than at admission. This represents worsening performance on a screening instrument. Both assessments were obtained during active psychosis and during antipsychotic treatment, and they were performed in different settings, one in an inpatient psychiatric unit shortly after involuntary admission and one in an outpatient clinic a month later. Attention, psychiatric state, medication effect, fatigue, and differences in testing conditions could each have contributed to the change, and none of these can be excluded retrospectively. We therefore report the scores and the affected domains without assigning a severity category to either assessment.

Domain-level performance showed impaired trail-making, cube copy, and clock drawing, delayed recall of 0/5 without benefit from cues, variable attention, reduced verbal fluency with intact repetition, relatively preserved abstraction, and orientation of 5/6 with disorientation to time. The three visuospatial and executive items depend on intact binocular vision and visuomotor scanning, and this patient had unilateral blindness of unclear etiology. The contribution of his visual impairment to those items cannot be quantified, and they should be interpreted with caution. The delayed recall finding does not depend on vision and is the more interpretable of the two. Its pattern, with no benefit from cueing, is suggestive of impaired encoding, although the MoCA cannot reliably distinguish impaired encoding from retrieval failure, and formal neuropsychological testing was not performed. At the time of this report the diagnostic lumbar puncture had not yet been performed, and the ultimate diagnosis and the disposition of his financial affairs remained undetermined.

## 3. Discussion

### 3.1. Late-Onset Erotomanic Delusions and the Case for Evaluating an Underlying Disorder

This case illustrates the co-occurrence, in a single patient, of erotomanic delusions, worsening cognitive screening performance, and structural ventriculomegaly. Late-onset psychosis without a prior psychiatric history should prompt systematic evaluation for secondary causes before a primary psychotic disorder is diagnosed. The differential in this age group includes neurodegenerative disease (Alzheimer’s disease, frontotemporal dementia, dementia with Lewy bodies), vascular cognitive impairment, normal pressure hydrocephalus, delirium, mood disorder with psychotic features, substance-induced psychosis, and late-onset schizophrenia-spectrum disorders [1,2]. In this patient the structural imaging abnormality, the vascular risk profile with imaging evidence of small-vessel disease, the multidomain cognitive findings, and the marked functional impairment together make an isolated primary psychiatric explanation unlikely. They do not, however, identify which secondary process was operating, and that is the central limitation of what follows.

### 3.2. Diagnostic Formulation and Differential Diagnosis

The admission working diagnosis was unspecified psychosis, with major neurocognitive disorder and delirium secondary to a medical condition on the differential. That formulation was appropriately cautious. Each alternative on it remains relevant for the reasons that follow.

Delusional disorder, erotomanic type, fits part of the picture. The erotomanic belief was fixed, thematically stable over weeks, and elaborated with supporting detail. But that diagnosis requires that functioning not be markedly impaired apart from the direct consequences of the delusion and that the presentation not be better explained by another condition. This patient had an illogical and circumstantial thought process at presentation, intermittent disorientation, impaired concentration, paranoid ideation toward staff and family, and multidomain abnormalities on cognitive screening, none of which are consequences of the erotomanic belief itself. The presentation is therefore described here as an erotomanic syndrome. The term delusional disorder, erotomanic type, appears only where the applicability of that diagnostic category is under discussion.

Delirium warrants closer attention than it is usually given once a delusional theme is identified, and this case has genuine delirium features. Orientation was intermittently impaired on examination. Collateral described waxing and waning clarity with periods of partial lucidity. Oral intake before admission was poor and uncertain. There was a mild leukocytosis with nonspecific urinalysis abnormalities. He received intramuscular haloperidol in the emergency department and was then started on quetiapine. Three considerations argue against delirium as the primary explanation. The course was approximately one month of progressive behavioral and cognitive change. Delirium characteristically develops over hours to days. The delusional content was thematically fixed and elaborated over weeks, with sustained goal-directed behavior organized around it, including the sale of a property and repeated financial transfers, which is not characteristic of delirium. And the presentation persisted at one-month outpatient follow-up, without an acute medical precipitant, with fixed delusions, disorientation to time, and worse cognitive screening performance. The persistence is the strongest of the three. No structured delirium instrument, such as the Confusion Assessment Method, was administered and documented, and attention was assessed clinically and on the MoCA without using a validated delirium measure. A superimposed delirium at the time of admission therefore cannot be excluded.

Vascular cognitive impairment deserves fuller consideration than the imaging emphasis on ventriculomegaly might suggest. The patient had hypertension, dyslipidemia, and chronic small-vessel ischemic disease documented on prior imaging and confirmed on both the admission CT and the MRI. That profile is sufficient on its own to account for executive dysfunction and behavioral change; cerebral small-vessel disease is a recognized contributor to late-life psychotic symptoms, and vascular pathology commonly coexists with both hydrocephalus and neurodegenerative disease in this age group. The visuospatial and executive items of the MoCA are confounded in this patient by unilateral blindness, so they cannot be used to argue that the cognitive profile points away from a frontal-subcortical process.

Psychotic disorder due to another medical condition and psychosis associated with a major neurocognitive disorder are the remaining alternatives, and this case cannot separate them, since the structural finding and the cognitive impairment support both. Dementia with Lewy bodies merits specific mention. The patient had psychosis, intermittent disorientation, and impaired performance on visual tasks, and he developed a bilateral resting tremor on low-dose risperidone. That tremor has two readings. It may represent medication-induced parkinsonism, which is common at this age even at 0.5 mg daily, or it may be an early parkinsonian feature of an underlying neurodegenerative process. The distinction carries direct treatment consequences given antipsychotic sensitivity in dementia with Lewy bodies, and it was not resolved during the period covered by this report. Very-late-onset schizophrenia-like psychosis is less likely given the prominent cognitive findings and the structural abnormality, although the international consensus criteria do not require their absence [2].

No cerebrospinal fluid or plasma biomarkers, no amyloid or tau imaging, and no formal neuropsychological testing were obtained. No neurodegenerative process was therefore characterized at any level beyond clinical screening.

### 3.3. Competing Explanatory Accounts for the Co-Occurrence of Findings

The temporal sequence in this case does not establish the direction of the relationship among the erotomanic syndrome, the cognitive impairment, and the ventriculomegaly. Two accounts of that relationship, and three possible statuses for the ventriculomegaly, are compatible with what was observed. They are not mutually exclusive (Figure 2).

In the first account, an underlying neurological or neurocognitive process, whether incipient hydrocephalus, cerebral small-vessel disease, or an evolving neurodegenerative disorder, gives rise to both the cognitive impairment and the erotomanic syndrome. Frontal-subcortical dysfunction, or medial temporal and parietal involvement, could lower the threshold for delusional misinterpretation of ambiguous social cues, such as a celebrity’s public statements or an unfamiliar caller, so that the erotomanic syndrome is an early behavioral marker of the underlying disorder rather than its cause [3,6]. The concurrent onset of cognitive and psychiatric change described by collateral is most consistent with this account.

In the second account, a primary very-late-onset psychotic syndrome occurred together with cognitive impairment that arose independently, for example, from this patient’s documented cerebrovascular disease. The two would then be coincident, with no causal link between them.

A separate question concerns the status of the ventriculomegaly itself, which may be a contributing neurological process, may be secondary to parenchymal volume loss, or may be an incidental structural finding of uncertain etiological significance. Nothing in this case establishes that the ventriculomegaly is a downstream structural consequence or a shared substrate of either account.

A further possibility, that a primary late-onset delusional disorder contributed to cognitive decline through chronic stress, sleep disruption, self-neglect, and psychotropic exposure, is described in the literature for longer illness courses. This mechanism is hypothesis-generating. The interval between symptom onset and presentation here was approximately one month, which is too short for that mechanism to account for the observed cognitive findings, and the collateral history places the cognitive and behavioral changes in the same window as the erotomanic beliefs. The two directions are therefore not equally supported by this patient’s trajectory.

Longitudinal studies that track psychiatric and cognitive symptoms alongside serial imaging in patients presenting with late-onset erotomanic delusions would be needed to determine which account, or combination, predominates.

### 3.4. Ventriculomegaly: Normal Pressure Hydrocephalus Versus Hydrocephalus Ex Vacuo

Differentiating idiopathic normal pressure hydrocephalus from hydrocephalus ex vacuo remains clinically challenging [9,10]. In ex vacuo ventriculomegaly, ventricular enlargement reflects parenchymal volume loss rather than a primary disturbance of cerebrospinal fluid dynamics. In this patient the distinction was not made. Gait was independent and largely preserved; there was no urinary incontinence; the recommended tap test was not performed; and the Evans index, the callosal angle, and features of disproportionately enlarged subarachnoid-space hydrocephalus were not measured [12]. The neurology formulation of borderline or early normal pressure hydrocephalus versus hydrocephalus ex vacuo with a superimposed neurocognitive disorder remains the most accurate description available for this case.

The diagnostic large-volume lumbar puncture, or tap test, recommended by neurology is the standard means of assessing shunt responsiveness. Its predictive performance is asymmetric. In the European iNPH Multicentre Study, a tap test defined by at least 5% improvement had a positive predictive value of 88% for outcome at 12 months but a negative predictive value of 18%, with sensitivity 52%, specificity 59%, and overall accuracy 53% [13]. A positive result therefore supports selection for shunting, whereas a negative result neither excludes the diagnosis nor rules out benefit from treatment, and that study found no correlation between the tap test result and outcome at 12 months. Cognitive response is also less consistently observed than gait response [13,15]. In this patient, whose presentation was dominated by cognitive and psychiatric features with little gait impairment, a negative tap test would not have eliminated diagnostic ambiguity. Formal cerebrospinal fluid biomarkers were not obtained, leaving open the possibility of a primary neurodegenerative process, of communicating hydrocephalus, of a vascular contribution, or of some combination. NPH frequently coexists with other neurodegenerative and cerebrovascular disease in older adults, and this comorbidity can limit cognitive recovery after cerebrospinal fluid diversion [9,16].

### 3.5. Financial Exploitation and Decisional Capacity: A Clinically Important Downstream Consequence

Irrespective of which account is correct, this case shows how an erotomanic syndrome and cognitive impairment can together translate into severe real-world harm. The patient executed major financial transactions, the sale of his home and the transfer of roughly $200,000, while holding fixed erotomanic beliefs and showing cognitive impairment on screening. No formal financial capacity instrument was administered, and no capacity determination was made at the time of the transactions. Capacity at the time of those transactions cannot be determined retrospectively from this record, and the concerns set out below are clinical.

Formal financial capacity assessment evaluates the ability to understand financial concepts, appreciate one’s own financial situation, reason about financial decisions, and communicate a choice [14]. This patient’s presentation, including his inability to appreciate that he was being defrauded, his illogical reasoning about the transactions, and his memory deficits, raises substantial concern for impairment across each of these domains. His professional background as a graduate-level economist and financial consultant establishes a high premorbid baseline, which strengthens the argument that his financial decisions during this period departed markedly from a lifelong pattern of competent financial management. It also illustrates that financial expertise does not protect against loss of financial decisional capacity once cognitive impairment and delusional thinking converge [7].

The pattern of financial loss in this case, large transfers to unknown individuals exploiting a delusional belief through social media impersonation, is strongly consistent with elder financial exploitation as defined in current surveillance criteria [17]. This pattern of self-victimization through delusion is distinct from the harm to third parties emphasized in most of the forensic erotomania literature [4,5]. As romance-scam and impersonation schemes conducted over telephone and social media become more prevalent, clinicians should be alert to this pattern in cognitively vulnerable older adults and should be aware of applicable mandatory-reporting obligations for suspected elder financial exploitation. Protective planning should begin with less restrictive alternatives, including representative payee arrangements, supported decision-making, and a durable power of attorney executed while capacity is retained [18]. Guardianship or other protective legal measures may need to be considered when formal capacity assessment demonstrates significant impairment and less restrictive alternatives are insufficient or infeasible [19]. Clinicians practicing in jurisdictions with a civil-law tradition, such as Puerto Rico, should be familiar with local statutory requirements for involuntary commitment [11], capacity determination, and guardianship, which can differ in relevant respects from common-law jurisdictions in the mainland United States.

### 3.6. Limitations

This case report has several limitations, and they bear directly on the central questions it raises. Formal neuropsychological testing beyond the MoCA was not performed, so the cognitive findings rest on a screening instrument administered twice, on both occasions during active psychosis and antipsychotic treatment, and in different settings. The visuospatial and executive items of that instrument are confounded by the patient’s unilateral blindness, and visual acuity in the remaining eye was not formally assessed at either session.

Quantitative radiological characterization was not obtained. The Evans index, the callosal angle, and features of disproportionately enlarged subarachnoid-space hydrocephalus were not measured during the admission, and the formal morphometric review that neurology recommended was not completed. Quantitative measurements were not performed because they would require review of the original imaging by a radiologist. The callosal angle also requires a specific coronal view that was not available. This constraint bears directly on the central diagnostic question of this report because the differentiation of normal pressure hydrocephalus from hydrocephalus ex vacuo depends substantially on these measurements.

The diagnostic lumbar puncture recommended by neurology had not been performed at the time of writing, so normal pressure hydrocephalus remained unconfirmed, and cerebrospinal fluid and plasma biomarkers for neurodegenerative disease were not obtained. No structured delirium instrument was administered, so a superimposed delirium at admission cannot be excluded. No formal financial capacity instrument and no standardized functional instrument were administered, and collateral was unavailable at follow-up, which limits comparison of baseline and follow-up activities of daily living. Extrapyramidal rating scales were not administered, and the onset of the bilateral resting tremor relative to antipsychotic exposure cannot be established.

As a single retrospective case, this report cannot establish causality or directionality among the erotomanic syndrome, the cognitive impairment, and the ventriculomegaly, and it cannot determine whether the ventriculomegaly was contributory, secondary, or incidental. The eventual legal and financial outcome of the case, including whether guardianship was pursued or the financial transactions were challenged, was not known at the time of writing.

## 4. Conclusions

Late-onset erotomanic delusions in an older adult without previous psychiatric history, particularly when accompanied by cognitive impairment or neurological findings, should prompt careful evaluation for an underlying neurocognitive or neurological disorder before attributing the presentation to an isolated primary psychiatric syndrome. In this patient an erotomanic syndrome, worsening cognitive screening performance, and ventriculomegaly disproportionate to cortical atrophy occurred together. The relationships among them were not established, and the competing accounts set out above remain open.

Evaluation in this setting should include structural neuroimaging with quantitative morphometric characterization where feasible, longitudinal cognitive assessment with formal neuropsychological testing, and, where a large-volume tap test is feasible, evaluation for normal pressure hydrocephalus, which remains one of the few potentially reversible contributors to this clinical picture. Where parkinsonian signs emerge, particularly during antipsychotic treatment, the possibility of an antipsychotic-sensitive neurodegenerative disorder should be considered and the treatment plan reviewed accordingly.

Impaired judgment in this setting can produce severe financial harm even in patients with high premorbid financial sophistication. Clinicians should therefore pair neuropsychiatric assessment with formal decisional capacity evaluation, should consider less restrictive protective alternatives first, and should consider guardianship or other protective legal measures when formal capacity assessment demonstrates significant impairment and less restrictive alternatives are insufficient or infeasible. Further longitudinal study is needed to clarify the relationship between late-onset erotomanic delusions and neurocognitive disorder and to determine how reliably ventriculomegaly in these patients distinguishes a treatable, hydrocephalus-related process from an irreversible neurodegenerative or cerebrovascular one.

## Figures and Tables

**Figure 1:**
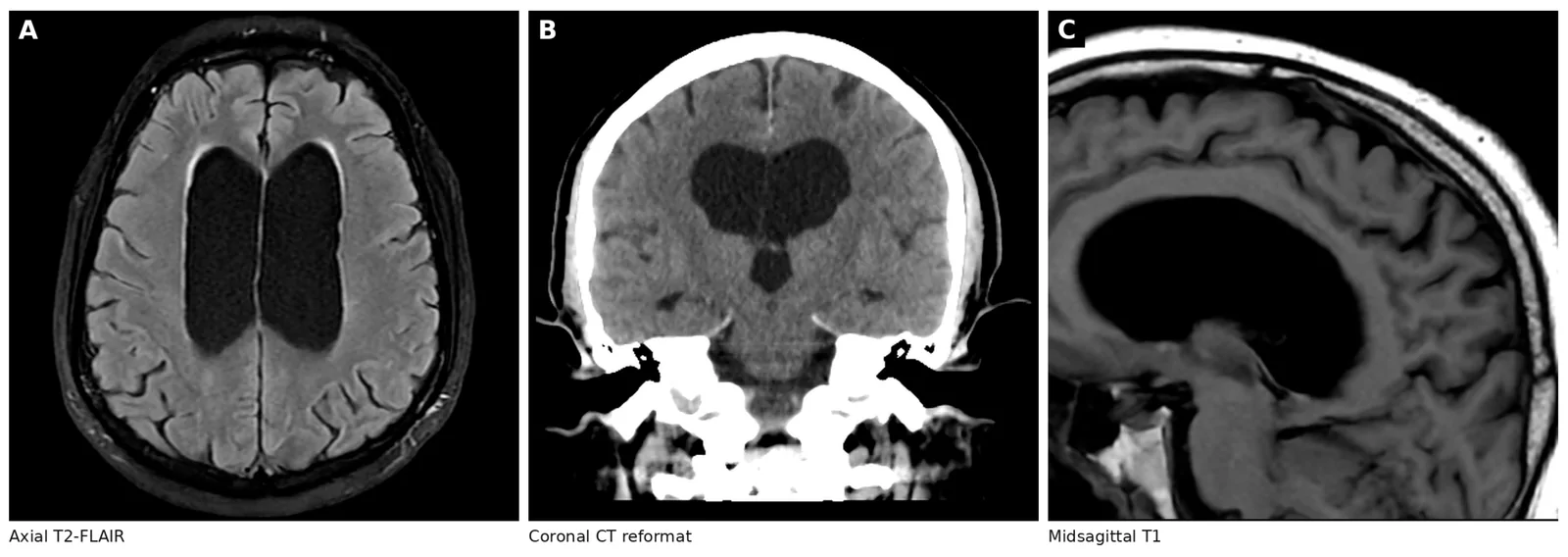
Neuroimaging at presentation. (**A**) Axial T2-FLAIR MRI showing marked lateral ventriculomegaly with periventricular hyperintensity, disproportionate to the degree of cortical sulcal prominence. (**B**) Coronal CT reformat showing the dilated lateral and third ventricles. (**C**) Midsagittal T1-weighted image showing the enlarged lateral ventricle and thinning of the corpus callosum. Quantitative morphometric measurements, including the Evans index and callosal angle, were not obtained.

**Figure 2:**
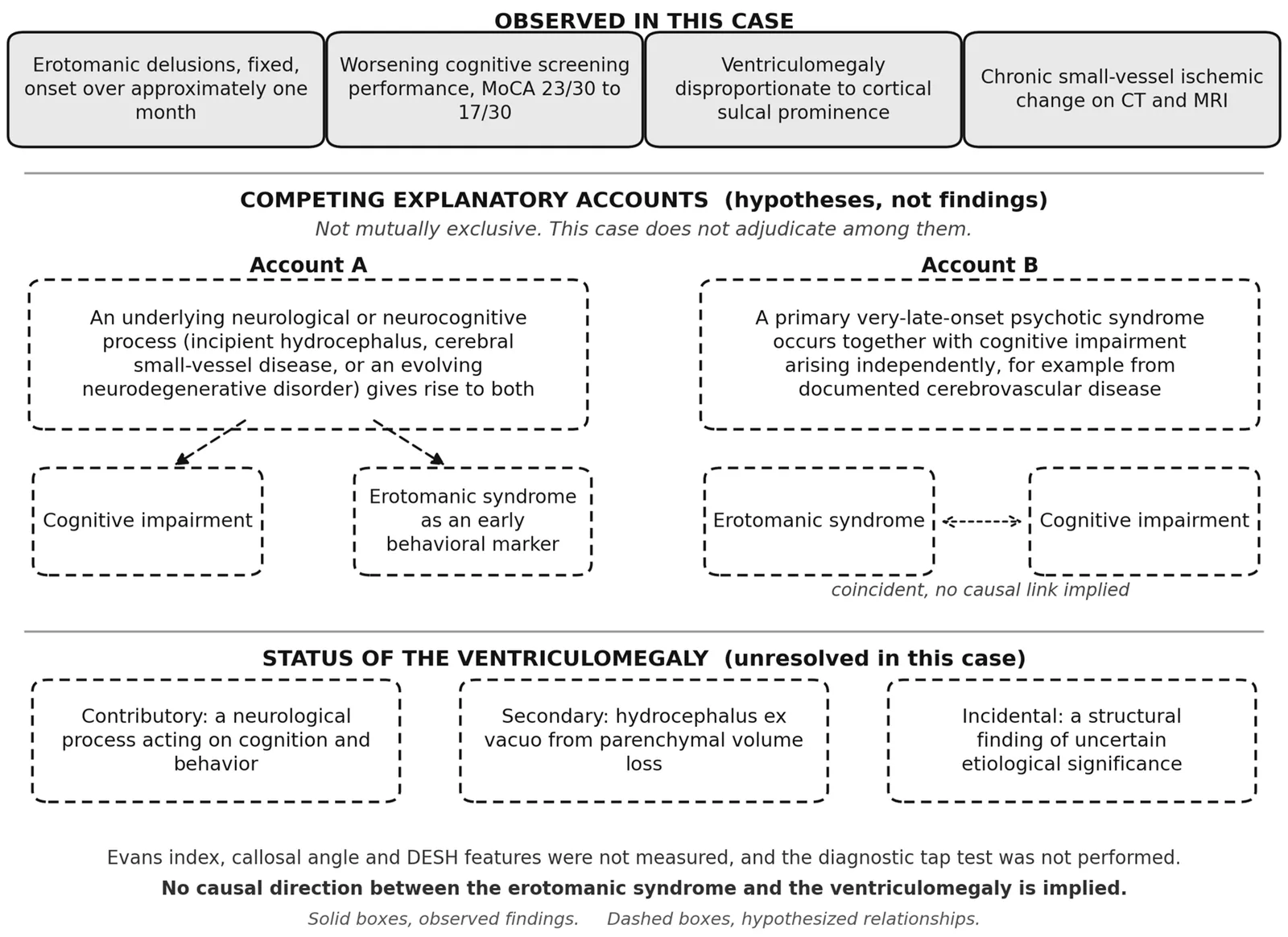
Competing explanatory accounts for the co-occurrence of findings in this case. The upper register shows what was observed. The lower register shows competing accounts of how those findings may be related, together with the three possible statuses of the ventriculomegaly. The accounts are not mutually exclusive, and this case does not adjudicate among them. No causal direction between the erotomanic syndrome and the ventriculomegaly is implied.

**Table 1:** Laboratory findings during hospitalization.

Category	Test	Result
Chemistry	Glucose	105 mg/dL
	Blood urea nitrogen (BUN)	22 mg/dL
	Creatinine	1.05 mg/dL
	Sodium	138 mmol/L
	Potassium	4.1 mmol/L
	Chloride	100 mmol/L
	CO2	26 mmol/L
	Calcium	9.5 mg/dL
	Albumin	4.5 g/dL
	AST	43 U/L
	ALT	43 U/L
	Alkaline phosphatase	55 U/L
	Total bilirubin	0.66 mg/dL
Hematology	WBC	12.20 × 10^3^/mcL
	RBC	4.76 × 10^6^/mcL
	Hemoglobin	14.4 g/dL
	Hematocrit	41.7%
	Platelets	217 × 10^3^/mcL
	Neutrophils	76.2%
	Lymphocytes	13.6%
Special chemistry	TSH	2.010 mIU/mL
	Vitamin B12	612 pg/mL
Serology	RPR	Non-reactive
Toxicology	THC/opiates/cocaine/fentanyl	Negative
Urinalysis	Appearance	Dark yellow, cloudy
	Blood	Trace
	Protein	100 mg/dL
	Ketones	Trace
	Leukocyte esterase	Trace
	Hyaline casts	11–20/lpf

Vital signs were stable throughout admission (blood pressure 100–121/58–79 mmHg, heart rate 60–86 bpm, temperature 36.5–36.8 °C, respiratory rate 16–20, oxygen saturation 99% on room air; weight 88.45 kg, height 180.34 cm).

## Data Availability

The de-identified clinical data supporting the findings of this case report are contained within the article. The original clinical records are not publicly deposited in order to protect patient confidentiality; further inquiries can be directed to the corresponding author.
